# Atypical Presentation of a Ruptured Anterior Communicating Artery Aneurysm With Subdural, Intraparenchymal, and Subarachnoid Hemorrhage

**DOI:** 10.7759/cureus.71788

**Published:** 2024-10-18

**Authors:** Nicholas Dietz, Aashka Sheth, Phil Ostrov, Edward Ham, Jersey Mettille, Heidi Koenig, Isaac Abecassis, Brian J Williams, Dale Ding

**Affiliations:** 1 Neurosurgery, University of Louisville Hospital, Louisville, USA; 2 Anesthesiology, University of Louisville Hospital, Louisville, USA

**Keywords:** anterior communicating artery, anuerysm rupture, intraparenchymal hemorrhage, subarachnoid hemorrhage, subdural hematoma

## Abstract

Anterior communicating artery (ACom) aneurysm, one of the most frequent types of intracranial aneurysm rupture, usually results in a subarachnoid hemorrhage (SAH) with intraventricular hemorrhage. We describe a case of an ACom aneurysm rupture with subarachnoid, intraparenchymal, and subdural hemorrhages (SDH) with midline shift in a 55-year-old woman. Decompressive craniectomy was performed to evacuate the SDH with subsequent microsurgical clipping of the aneurysm. Postoperative angiogram showed occlusion of the ACom aneurysm without residual flow. Mechanisms to explain multimodal bleeding with SAH, intraparenchymal hemorrhage, and SDH include adhesion of aneurysm to arachnoid membrane, high pressure hemorrhage, and spontaneous laceration of the arachnoid membrane. Management of multifocal hemorrhage pattern including SDH after ACom aneurysm rupture is dependent on anatomical and radiographic features as well as the clinical condition of the patient on admission. Decompressive craniectomy is a suitable treatment option for patients with concurrent spontaneous SAH, intraparenchymal hemorrhage, and SDH.

## Introduction

Anterior communicating artery (ACom) aneurysm rupture represents the most common form of intracranial aneurysm rupture, observed in 23-40% of cases [1]. Aneurysm rupture results in subarachnoid hemorrhage (SAH) in 80% of cases and intraventricular hemorrhage in 17% of cases, but rarely results in subdural hematoma (SDH) or intraparenchymal hemorrhage [2]. Risk of ACom aneurysm rupture is based on anatomical and hemodynamic characteristics as well as certain modifiable and non-modifiable risk factors including, but not limited to, smoking, pulsatility index, and asymmetry of A1 segments [1,2]. A smaller A1-A2 angle of ACom is associated with aneurysm due to the higher distribution of hemodynamic force [1]. Several techniques used to treat and repair ACom aneurysm are endovascular coiling, stent-assisted embolization, and microsurgerical clipping. Treatment choice is dependent on several factors such as clinical assessment, anatomical features of the artery, presence of intracerebral hematoma, age, blood thinners, and associated comorbidities [3].

SDH accompanying aneurysm rupture is rare but most commonly reported with rupture of posterior communicating artery aneurysms [4]. Clinical presentation of SDH associated with aneurysm rupture involves headaches followed by seizures with quick progression to obtundation and a Glasgow Coma Scale (GCS) of less than 6. [5]. We report a unique case of ACom aneurysm rupture that presented with large hemispheric SDH and intraparenchymal hemorrhage.

## Case presentation

A 55-year-old woman with a past medical history of hypertension presented with severe headache to an outside hospital. Within two hours, the patient became comatose and unresponsive, requiring intubation in the field by EMS. There was no family history of aneurysm, smoking, or drug use, and no inciting trauma. On admission, temperature was 36.7 °C, heart rate was 165 bpm, and blood pressure was labile, measuring at 240/160 with systolic pressures ranging from 80-240 mmHg. Her blood pressure was controlled immediately with a Nicardipine drip to a goal of 130-150 systolic blood pressure to prevent rebleed. Additionally, the target mean arterial pressure (MAP) of 70-80 mmHg and heart rate of 80-100 bpm were used to prevent secondary injury to the brain and heart. The patient’s initial GCS was E1VTM1=GCS 3T. Initial labs showed the following levels: arterial blood gas with pH of 7.31, partial pressure of carbon dioxide as 38.7 mmHg, sodium as 137 mmol/L, hemoglobin as 15.0 g/dL, hematocrit as 46.1%, and platelets 251 mcL.

**Figure 1:**
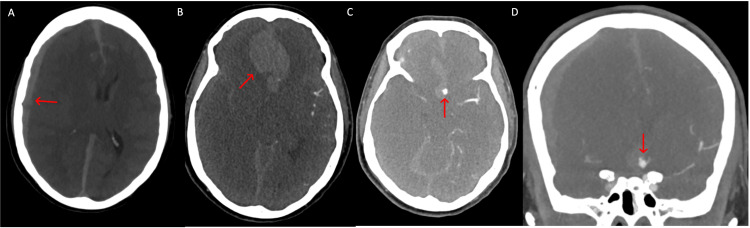
A: Preoperative axial CT demonstrating interhemispheric and convexity subdural hematoma >8 mm (red arrow) with mass effect and bilateral acute subarachnoid hemorrhage, B: Preoperative axial CTA demonstrating large bifrontal intraparenchymal hemorrhage (red arrow), C: Preoperative axial CTA demonstrating 4 mm left sided anterior communicating artery aneurysm (red arrow) with surrounding intraparenchymal hemorrhage, tentorial subdural hemorrhage, and bilateral subarachnoid hemorrhage, D: Preoperative coronal CTA with left convexity subdural hematoma, anterior communicating artery aneurysm (red arrow), and surrounding intraparenchymal hemorrhage

The patient’s exam on arrival showed non-reactive pupils, absent corneal reflex, absent gag reflex, and absent cough reflex. Post intubation, computed tomography angiography showed a five mm left ACom artery aneurysm with an eight mm SDH in the right hemispheric convexity and associated hemorrhage in the right medial frontal lobe (Figure 1). There was associated vasogenic edema in the bilateral frontal lobes, one cm leftward midline shift, and effacement of the basal cisterns. The patient was diagnosed with an SAH; World Federation of Neurosurgical Societies (WFNS) grade V; Hunt and Hess grade 5; and modified Fisher grade 4. The patient was given 60 mg of Nimodipine every 4 hours and maintenance IV fluids, including 125 cc per hour of normal saline. The patient was medically stabilized and emergently taken to the operating room for left-sided decompressive craniectomy, hematoma evacuation, and aneurysm clipping.

Intraoperatively, a left-sided decompressive craniectomy was performed to relieve the mass effect. After exposure of the left recurrent artery of Heubner (RAH), left A2, right A1, right RAH, and right A2, a large compressive coagulated blood clot was observed grossly underlying the dura. Anteriorly and superiorly, a gross examination of the brain demonstrated vascular congestion and bruising, presumably from the cortical hemorrhage. Care was taken to appreciate increased bleeding from the removal of the clot, the tamponade effect, and any ensuing oedema or inflammation. Levetiracetam was administered at 500 mg twice daily for seizure prophylaxis. Mannitol at 1 g/kg was also administered to reduce the mass effect and herniation potential from cerebral swelling. Nicardipine, previously administered, drops the diastolic and systolic pressures but can be cleared quicker than Labetalol, allowing for greater minute-to-minute control [6]. Because inhaled anesthetics increase cerebral blood flow and intracranial pressure, total IV anesthesia (propofol) was primarily used.

Subsequent goals of surgery were to secure and clip the ACom aneurysm under microscopic dissection. After dissection of the sylvian fissure, release of cerebrospinal fluid was achieved through the opening of the opticocarotid cistern and lamina terminalis. Relaxation of the brain was observed, allowing improved visualization of the neurovascular anatomy. A large left ACom aneurysm was identified with a daughter sac, and ipsilateral A1 and A2 vessels were dissected off the aneurysmal mass to allow visualization and preservation during the clipping. A 6.5 mm upward curved clip was applied parallel to the left A1-A2 junction. Indocyanine green solution and a doppler test were used to confirm appropriate clipping and occlusion of the aneurysm with patency of surrounding vessels.

**Figure 2:**
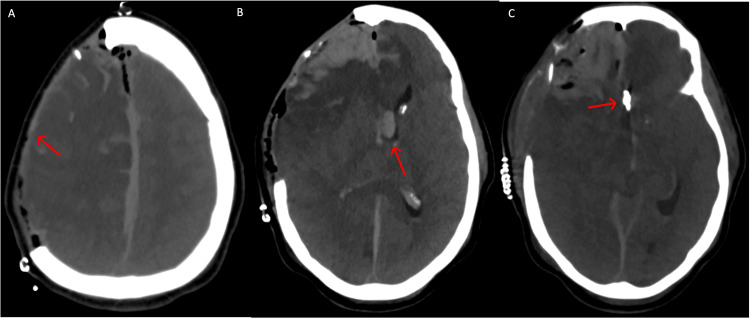
A: Postoperative axial CTH showing evacuated subdural hematoma with right-sided decompressive craniectomy (red arrow), B: Postoperative axial CTH depicting intraventricular hemorrhage (red arrow) and frontal intraparenchymal hemorrhage, C: Postoperative axial CTH demonstrating clipping of the left anterior communicating artery aneurysm (red arrow)

The patient was left intubated after the surgery, given her Glasgow Coma Scale (GCS) of 3(totally unresponsive)T. Propofol was continued postoperatively in transition to the ICU, where the patient was maintained for stable hemodynamics, blood pressure, and intracranial pressure, limiting swings pressure. Immediately postoperatively, the patient improved on examination including reactivity of pupils and withdrawal to stimulation in extremities. An angiogram confirmed a completely occluded left ACom aneurysm with bilateral ACom A1 and A2 segments patent and normal flow (Figure 2).

## Discussion

Intracranial aneurysm rupture with accompanying SDH rarely co-present, with an incidence rate of less than 3% of cases [7,8]. Implications of concurrent presentation suggest potential related trauma, arachnoid membrane tear, or an additional source of bleeding. ACom aneurysms have a particularly low rate of concurrent SDH [9]. The present case describes an ACom aneurysm rupture with SAH and accompanying acute SDH and intraparenchymal hemorrhage with mass effect treated by decompressive craniectomy and microsurgical clipping.

When SDH from aneurysmal rupture occurs, it is most frequently caused by rupture of internal carotid-posterior communicating artery aneurysms (30%) or middle cerebral artery aneurysms (27%) [9,10]. Isolated SDH from ruptured aneurysm without SAH has also rarely been described and is related to poor prognosis with high morbidity and mortality (~70%) [11]. Tsukagoshi and colleagues report SAH and thin acute-on-chronic-appearing SDH resulting from ACom aneurysm rupture in a 49-year-old female treated with endovascular coiling only, with no surgical intervention [8]. SDH is most frequently a consequence of traumatic brain injury, but several mechanisms have been proposed to explain SDH following spontaneous SAH [7,12,13]. An adherent arachnoid membrane could penetrate the subdural space due to arterial pressure from aneurysm rupture and consequently track to the convexity location [12, 13]. Intracerebral hemorrhage (also present in the current case) could rupture the cortex, lacerate the arachnoid membrane, and reach the subdural space [7]. When nontraumatic spontaneous SDH is observed, clinicians should be vigilant for ruptured cortical aneurysm [9]. Patients who present with acute SDH generally have “acute onset neurological impairment” that can be mistaken for head trauma or stroke [9]. Computed tomography angiography should be obtained prior to surgery to determine the best treatment strategy [13]. SDH secondary to aneurysm rupture may be accompanied by intracerebral hematoma and/or SAH, which can help healthcare providers differentiate from SDH caused by traumatic brain injury [9]. Frequent follow-ups with imaging to detect changes in volume are recommended for patients with ACom aneurysms to monitor aneurysm progression [8].

Subdural bleeding with significant mass effect and midline shift after aneurysm rupture is a life-threatening condition and requires immediate surgical management. While surgical clipping may be indicated for emergent patients, decompressive craniectomy has been shown to be a beneficial treatment option in patients with SAH and intracerebral hematoma [14, 15, 16]. Patients who are in stable hemodynamic condition have a higher chance of recovering in good condition after rapid surgical decompression [14]. Otani et al. report favorable outcomes following decompressive craniectomy in 41.7% of patients who presented with SAH and SDH [15]. Similarly, Guresir et al. found that 26.6% of patients with poor-grade SAH had favorable outcomes following decompressive craniectomy [16]. Their results indicated that decompressive craniectomy may be necessary regardless of whether the patient suffers from bleeding, infarction, or brain swelling. A systematic literature review found that 49% of patients with poor-grade SAH achieved favorable functional long-term outcomes following decompressive craniectomy [17]. Decompressive craniectomy can lower intracranial pressure regardless of aneurysm location. In the rare event of SDH and SAH presenting concurrently after ACom aneurysm rupture, we have demonstrated that decompressive craniectomy is an appropriate surgical measure and is sufficient for subdural evacuation, dissection of the Sylvian fissure, and subsequent aneurysm clipping. 

Treatment of ACom aneurysm rupture is dependent on the clinical condition of patients upon admission. Patients with World Federation of Neurosurgeons (WFNS) grade 1-5, equal and reactive pupils, and without significant brain ischemia are treated actively [3]. Navratil and colleagues outline a protocol to decide whether aneurysms should be treated via microsurgery or endovascular treatment [3]. Patients who meet the criteria for active treatment should be treated via microsurgery if their aneurysm is projecting anteriorly or inferiorly from ACom. If there is a risk of occlusion of branching vessels by endovascular treatment and the patient is younger than 60 years with minor comorbidities and not on blood thinners, microsurgery should be considered. Patients who meet the criteria for active treatment and have posteriorly projecting aneurysms may best be treated endovascularly [3]. However, for patients with aneurysms with narrow necks, older than 60 years, significant comorbidities, or use of blood thinners, endovascular treatment may be best [3]. Criteria for emergency surgical intervention include dropping at least two points on the GCS, pupil abnormalities, and severe radiologic evidence (SDH thickness > 10 mm or midline shift > 5 mm) [9]. Microsurgery treatment of ACom aneurysms can be difficult due to the variable anatomical location of the artery, deep between both frontal lobes [3]. Additionally, stent placement can change the vascular geometry of the artery and increase hemodynamic stress [1]. Clipping can be difficult if an aneurysm is projecting between both A2 branches, so the treatment method is chosen on a case-by-case basis [3]. Age greater than 60 years, presence of hypertension, alteration of consciousness during SAH onset, Hunt and Hess and WFNS grades 3-5 on admission, A1 segment hypoplasia, and preoperative intraventricular hemorrhage are associated with unfavorable outcomes 6 months post operation for patients who undergo surgical clipping [18].

## Conclusions

ACom aneurysm may rarely present with concurrent SAH, SDH, and intraparenchymal hemorrhage. Treatment of SDH following ACom aneurysm rupture is dependent on anatomical and radiographic features as well as clinical condition of the patient on admission. Decompressive craniectomy is a reasonable treatment option in patients presenting with multimodal bleeding patterns with significant mass effect.
